# Phase Ib/II trial of talimogene laherparepvec alone and with pembrolizumab in advanced solid tumors with liver metastases and hepatocellular carcinoma

**DOI:** 10.1093/oncolo/oyae203

**Published:** 2025-03-28

**Authors:** Joel Randolph Hecht, Arjun Oberoi, Elena Garralda Cabanas, Hong Jae Chon, Antonia Digklia, Sylvie Rottey, Miguel Martin Jimenez, Marya Chaney, Jane Hippenmeyer, Tatiana Lawrence, Kate Liu, Ali Hamidi, Jason Chesney

**Affiliations:** UCLA Jonsson Comprehensive Cancer Center, Santa Monica, CA 90404, United States; Medical Oncology Department, Vall d´Hebron Institute of Oncology, Barcelona 08035, Spain; Department of Medicine, Vall d’Hebron University Hospital, Barcelona 08035, Spain; Department of Medicine, Universitat Autònoma de Barcelona, Barcelona 08193, Spain; Medical Oncology Department, Vall d´Hebron Institute of Oncology, Barcelona 08035, Spain; CHA Bundang Medical Center, CHA University, Bundang-Gu 13496, South Korea; Department of Oncology, Lausanne University Hospital, Lausanne 1011, Switzerland; Department of Oncology, Ghent University Hospital, Ghent 9000, Belgium; Department of Medical Oncology, Gregorio Marañón General University Hospital, Madrid 28007, Spain; Merck & Co., Inc., Rahway, NJ 07065, United States; Amgen Inc., Thousand Oaks, CA 91320, United States; Amgen Inc., Thousand Oaks, CA 91320, United States; Amgen Inc., Thousand Oaks, CA 91320, United States; Amgen Inc., Thousand Oaks, CA 91320, United States; James Graham Brown Cancer Center, University of Louisville, Louisville, KY 40202, United States

**Keywords:** hepatocellular carcinoma, liver metastasis, pembrolizumab, solid tumor, talimogene laherparepvec

## Abstract

**Background:**

Newer effective therapies are needed for patients with solid tumors with liver metastases and unresectable hepatocellular carcinoma (HCC).

**Methods:**

Part 1 (dose exploration) evaluated intrahepatic talimogene laherparepvec (T-VEC) injection in group A (non-HCC liver metastases) and group B (HCC). Cohorts 1-4 received T-VEC monotherapy; cohorts 5 and 6 received T-VEC+pembrolizumab. Part 2 (dose expansion) evaluated intrahepatic or intratumoral T-VEC+pembrolizumab in non-HCC solid tumors. The primary endpoints were dose-limiting toxicities (DLTs) in part 1; objective response rate (ORR) per modified irRC-RECIST and safety in part 2.

**Results:**

Part 1 enrolled 28 and 46 patients to receive T-VEC and T-VEC+pembrolizumab, respectively. Three patients reported DLTs (T-VEC, *n* = 2 grade 3 abdominal pain and aspartate transaminase increase; T-VEC+pembrolizumab, *n* = 1 grade 3 cholestatic hepatitis). ORR (secondary endpoint) with T-VEC was 0%; ORR (95% CI) with T-VEC+pembrolizumab was 8.3% (1.0, 27.0) for non-HCC and 13.6% (2.9, 34.9) for HCC. Part 2 enrolled 53 patients; ORR (95% CI) was 0% (0.0, 30.8)-20.0% (0.5, 71.6) across 5 tumor types, with 16.7% (95% CI: 3.6, 41.4) for triple-negative breast cancer with the largest sample size (*n* = 18). Safety findings were consistent with the therapies administered.

**Conclusions:**

Limited efficacy across tumor types evaluated limit further evaluation of intrahepatic T-VEC+pembrolizumab in this patient population.

**ClinicalTrials.gov Identifier:**

NCT02509507.

## Teaser

This study is the first investigation of intrahepatic T-VEC injection in patients with liver lesions due to metastatic solid tumors or hepatocellular carcinoma.

**Lessons learned**

- Talimogene laherparepvec (T-VEC) combined with pembrolizumab did not demonstrate evidence of additional efficacy in the treatment of solid tumors with liver metastases nor for primary hepatocellular carcinoma (HCC).
- T-VEC (administered via intrahepatic or intratumoral injection) alone or in combination with pembrolizumab is not a viable strategy for the treatment of primary HCC and advanced solid tumors with and without liver metastases.

## Discussion

Intrahepatic injection of oncolytic viruses has been considered an alternative approach for treatment of liver tumors over localized therapies such as liver resection and traditional interventional radiology. This study represents the first investigation of intrahepatic talimogene laherparepvec (T-VEC) injection in patients with liver lesions due to metastatic solid tumors or HCC. It was hypothesized that intrahepatic T-VEC injection would be tolerable and safe and would provide control of injected and uninjected hepatic and non-hepatic lesions, and addition of pembrolizumab would further enhance the local and systemic oncolytic effect of T-VEC.

Dose-limiting toxicities (DLTs) with intrahepatic T-VEC monotherapy were reported in 2 patients, including grade 3 abdominal pain (*n* = 1) and grade 3 AST increase (*n* = 1). In part 1, T-VEC+pembrolizumab was associated with 1 DLT of grade 3 cholestatic hepatitis. Overall, 3 patients in part 1 experienced hepatic hemorrhage during the study. One patient experienced fatal hepatic hemorrhage the day following protocol mandated liver biopsy followed by T-VEC injection to the same site. Another patient developed grade 2 hepatic hemorrhage during cycle 1 following a liver biopsy. This patient then developed grade 4 hepatic hemorrhage following T-VEC injection in cycle 2. A third patient had grade 3 hepatic hemorrhage following liver biopsy and T-VEC administration in the same tumor location, which resolved with supportive care. In these cases, hepatic hemorrhage was not considered by investigators to be related to T-VEC, but related to the study procedure of liver biopsy followed by T-VEC injection. The study protocol was amended to discontinue procedures of intrahepatic T-VEC injections and liver biopsies.

The remaining treatment-emergent adverse events for both parts were generally consistent across cohorts and aligned with the known safety profiles of either agent.

Efficacy assessments in part 1 showed no responses with T-VEC monotherapy and limited responses with T-VEC+pembrolizumab. In cohorts with non-HCC liver metastasis, T-VEC+pembrolizumab yielded an ORR of 8.3% (95% CI: 1.0, 27.0), DCR of 25.0% (95% CI: 9.8, 46.7), and median PFS and OS of 2.0 months (95% CI: 1.9, 6.2) and 7.8 months (95% CI: 4.4, 14.2), respectively. In HCC cohorts, T-VEC+pembrolizumab resulted in an ORR of 13.6% (95% CI: 2.9, 34.9), DCR of 40.9% (95% CI: 20.7, 63.6), and median PFS and OS of 8.1 months (95% CI: 3.2, 13.2), and 12.8 months (95% CI: 6.8, 29.5), respectively. In part 2, T-VEC+pembrolizumab demonstrated ORRs ranging from 0% in the CRC arm to 20.0% in the BCC arm. The only arm to enroll more than 10 patients was TNBC (*n* = 18), which demonstrated an ORR of 16.7% (95% CI: 3.6, 41.4), with 2 CRs and 1 PR, DCR of 22.2%, median PFS of 2.9 months (95% CI: 1.2, 10.2), and OS of 10.2 months (95% CI: 2.7, 27.2) (Figure 3). Overall, the efficacy data do not support further evaluation of T-VEC+pembrolizumab in this patient population.

## Trial Information

**Table:** 

Disease	Colorectal adenocarcinoma (CRC), hepatocellular carcinoma (HCC), breast cancer, gastroesophageal cancer, melanoma, non-small cell lung carcinoma (NSCLC), renal cell cancer (RCC), basal cell carcinoma (BCC), and cutaneous squamous cell carcinoma (CSCC)
Stage of disease/treatment	Locally advanced/metastatic; liver lesions
Prior therapy	At least 1 prior regimen for patients with non-HCC liver metastasis; prior antiviral therapy for patients with HCC
Type of study	Phase Ib/II, 3+3 (monotherapy cohort); modified toxicity probability interval up-and-down design (combination therapy cohort)
Primary endpoint	DLT in part 1; ORR per modified irRC-RECIST and safety (patient incidence, for each tumor type arm, of adverse events including DLTs) in part 2
Secondary endpoints	Efficacy (ORR [for part 1], BOR, DRR, DOR, DCR, PFS, and OS) and safety

## Additional details of endpoints or study design

This multicenter, open-label, phase Ib/II basket trial enrolled patients with primary HCC and advanced solid tumors from February 2016 to July 2023 and was conducted at 22 centers in Australia, Europe, South Korea, and the US. It was designed to evaluate the safety of intrahepatic injection of T-VEC into liver tumors, alone and in combination with systemic intravenous administration of pembrolizumab, in patients with non-HCC liver metastases from breast cancer, colorectal cancer, gastroesophageal cancer, melanoma, non-small cell lung cancer, and renal cell cancer in part 1 group A, and patients with HCC with and without viral hepatitis in part 1 group B (viral hepatitis only applicable in combination setting), and to evaluate the efficacy and safety of intratumoral and/or intrahepatic T-VEC in combination with systemic pembrolizumab in patients with advanced triple-negative breast cancer (TNBC), hormone receptor-positive breast cancer (HRBC), CRC, CSCC, and BCC in part 2 group A and patients with HCC with and without viral hepatitis in part 2 group B. Part 2 group B did not open for enrollment.

Following the events of hepatic hemorrhage, study screening and enrollment activities were put on a temporary halt. The temporary halt then led to a close of further study enrollment and a subsequent protocol amendment (dated 26 October 2021) that discontinued all protocol-specified transcutaneous intrahepatic T-VEC injections and transcutaneous liver biopsies. Patients already enrolled (and on study treatment) were reconsented and permitted to continue treatment with the non-hepatic route of T-VEC injections, which could continue for patients who derived clinical benefit as per the discretion of the investigator.

The study comprised a safety follow-up visit 30 (+7) days after the last dose of T-VEC for the monotherapy cohorts or 30 (+7) days after the last dose of T-VEC or pembrolizumab, whichever was later, for the combination treatment. All patients who permanently discontinued study drug for any reason other than withdrawal of consent were assessed for survival, T-VEC-related adverse events, and initiation of additional antitumor therapy every 12 weeks (±28 days) following the safety follow-up visit until death, patient withdrawal, or up to approximately 24 months after the date of the last patient enrolled (part 1). Patients in cohorts 5 and 6 were followed for approximately 24 months after the last patient enrolled in their cohort in part 1, or approximately 24 months after the last patient enrolled with their tumor type in part 2, whichever was later. In part 2, patients were followed for up to approximately 24 months after the date of the last patient enrolled in that tumor cohort. The data cutoff for the final analysis was 18 August 2023. The trial was conducted in accordance with the International Council for Harmonization Good Clinical Practice guidelines and the principles of the Declaration of Helsinki. The protocol and amendments were approved by the Institutional Review Board/Independent Ethics Committee at each participating site (Table 1). All patients provided written informed consent.

**Table 1.** List of institutional review board (IRB) or independent ethics committee (IEC) for all sites that enrolled patients.

Site name	IRB/IEC name	IRB/IEC address	Regional IRB/IEC name	Regional IRB/IEC address
Melanoma Institute Australia	South Western Sydney Local Health District Human Research Ethics Committee	Elizabeth Street, Level 2 UNSW Clinical School, Liverpool Hospital, Liverpool, NSW, 2170, Australia		
Tasman Oncology Research	Bellberry Limited, Human Research Ethics Committee	123 Glen Osmond Road, Bellberry Office SA, Eastwood, SA, 5063, Australia		
Landeskrankenhaus Salzburg	Ethikkommission fuer das Bundesland Salzburg	Sebastian-Stief-Gasse 2, Salzburg, 5010, Austria		
Universitair Ziekenhuis Gent	Universitair Ziekenhuis Gent—Ethisch Comite	Corneel Heymanslaan 10, Ingang 75, tweede verdieping, Gent, 9000, Belgium	Commission d Ethique Biomedicale Hospitalo-Facultaire de l Universite catholique de Louvain	Promenade de l’Alma 51, Boite B1.43.03, Brussels, 1200, Belgium
Universitair Ziekenhuis Gent	Commission d Ethique Biomedicale Hospitalo-Facultaire de l Universite catholique de Louvain	Promenade de l’Alma 51, Boite B1.43.03, Brussels, 1200, Belgium	Commission d Ethique Biomedicale Hospitalo-Facultaire de l Universite catholique de Louvain	Promenade de l’Alma 51, Boite B1.43.03, Brussels, 1200, Belgium
Universitair Ziekenhuis Antwerpen	Universitair Ziekenhuis Antwerpen—Ethisch Comite	Drie Eikenstraat 655, Universitair Ziekenhuis Antwerpen, Edegem, 2650, Belgium	Commission d Ethique Biomedicale Hospitalo-Facultaire de l Universite catholique de Louvain	Promenade de l’Alma 51, Boite B1.43.03, Brussels, 1200, Belgium
Universitair Ziekenhuis Antwerpen	Commission d Ethique Biomedicale Hospitalo-Facultaire de l Universite catholique de Louvain	Promenade de l’Alma 51, Boite B1.43.03, Brussels, 1200, Belgium	Commission d Ethique Biomedicale Hospitalo-Facultaire de l Universite catholique de Louvain	Promenade de l’Alma 51, Boite B1.43.03, Brussels, 1200, Belgium
Kreiskliniken Reutlingen—Klinikum am Steinenberg	Ethik-Kommission bei der Landesaerztekammer Baden-Wuerttemberg	Jahnstrase 40, Stuttgart, 70597, Germany		
Kreiskliniken Reutlingen—Klinikum am Steinenberg	Ethikkommission der Medizinischen Fakultaet am Universitaetsklinikum	Gartenstrasse 47, Tuebingen, 72074, Germany		
Severance Hospital Yonsei University Health System	Severance Hospital Institutional Review Board	134 Shinchon-dong, Seodaemun-gu, Department of Internal Medicine, Seoul, 120-752, South Korea		
Cha Bundang Medical Center, Cha University	IRB of Cha Bundang Medical Center Cha University	59, Yatap-ro, Bundang-gu, Institutional Review Board, CHA Bundang Medical Center, CHA University, Seongnam-si, Gyeonggi-do, 13496, South Korea		
Hospital General Universitario Gregorio Marañon	CEIC Hospital General Universitario Gregorio Marañon	Calle del Doctor Esquerdo 46, Pabellon de Gobierno Planta baja, Madrid, Madrid, 28007, Spain	CEIC Hospital General Universitario Gregorio Marañon	Calle del Doctor Esquerdo 46, Pabellon de Gobierno Planta baja, Madrid, Madrid, 28007, Spain
Hospital Universitario Madrid Sanchinarro	CEIC Grupo Hospital de Madrid	Avenida Monteprincipe 25, Hospital Universitario Monteprincipe Edificio Docente Facultat de Medicina, Boadilla del Monte, Madrid, 28660, Spain	CEIC Hospital General Universitario Gregorio Marañon	Calle del Doctor Esquerdo 46, Pabellon de Gobierno Planta baja, Madrid, Madrid, 28007, Spain
Hospital Clinic i Provincial de Barcelona	CEIC Hospital Clinic i Provincial de Barcelona	Carrer de Villarroel 170, Farmacia EECC Esc 6B Sotano, Barcelona, Cataluña, 08036, Spain	CEIC Hospital General Universitario Gregorio Marañon	Calle del Doctor Esquerdo 46, Pabellon de Gobierno Planta baja, Madrid, Madrid, 28007, Spain
Hospital Universitari Vall d Hebron			CEIC Hospital General Universitario Gregorio Marañon	Calle del Doctor Esquerdo 46, Pabellon de Gobierno Planta baja, Madrid, Madrid, 28007, Spain
Kantonsspital Winterthur	Comite Departemental dEthique de Medecine Interne et Medecine Communautaire	Rue Gabrielle-Perret-Gentil 4, Geneva 14, 1211, Switzerland	Commission cantonale d’etique de la recherche	Rue Adrien-Lachenal 8, Geneve, 1207, Switzerland
Kantonsspital Winterthur	Kantonale Ethikkommission Zuerich	Stampfenbachstrasse 121, Zuerich, 8090, Switzerland	Commission cantonale d’etique de la recherche	Rue Adrien-Lachenal 8, Geneve, 1207, Switzerland
Hopitaux Universitaires de Geneve	Commission cantonale d’etique de la recherche	Rue Adrien-Lachenal 8, Geneve, 1207, Switzerland	Commission cantonale d’etique de la recherche	Rue Adrien-Lachenal 8, Geneve, 1207, Switzerland
Centre Hospitalier Universitaire Vaudois	Commission cantonale d’ethique de la recherche sur l’etre humain	Avenue de Chailly 23, Lausanne, 1012, Switzerland	Commission cantonale d’etique de la recherche	Rue Adrien-Lachenal 8, Geneve, 1207, Switzerland
Universitaetsspital Zuerich	Comite Departemental dEthique de Medecine Interne et Medecine Communautaire	Rue Gabrielle-Perret-Gentil 4, Geneva 14, 1211, Switzerland	Commission cantonale d’etique de la recherche	Rue Adrien-Lachenal 8, Geneve, 1207, Switzerland
Universitaetsspital Zuerich	Kantonale Ethikkommission Zuerich	Stampfenbachstrasse 121, Zuerich, 8090, Switzerland	Commission cantonale d’etique de la recherche	Rue Adrien-Lachenal 8, Geneve, 1207, Switzerland
University of Louisville James Graham Brown Cancer Center	University of Louisville IRB Human Subjects Protection Program Office	501 East Broadway, MedCenter One, Suite 200, Louisville, KY, 40202, USA		
Washington University School of Medicine, Center for Advanced Medicine	Washington University School of Medicine, Human Research Protection Office	660 South Euclid Avenue, Box 8089, St Louis, MO, 63110, USA		
University of California Los Angeles	University of California Los Angeles Office of the Human Research Protection Program	11 000 Kinross Avenue, Suite 211 Box 951694, Los Angeles, CA, 90095-1694, USA		
University of Texas MD Anderson Cancer Center	The University of Texas MD Anderson Cancer Center Institutional Review Board	7007 Bertner Avenue, Unit 1637, Houston, TX, 77030, USA		
University of Pittsburgh	University of Pittsburgh Human Research Protection Office	3500 5th Avenue, Heiber Building, Main Office, Suite 106, Pittsburgh, PA, 15213, USA		
HonorHealth Research Institute	Western Institutional Review Board	1019 39th Avenue Southeast, Suite 120, Puyallup, WA, 98374, USA		

Key eligibility criteria in part 1 included age ≥18 years; liver metastases from non-HCC (group A), including breast adenocarcinoma, CRC, gastroesophageal cancer, melanoma, non-small-cell lung cancer, or clear cell renal cell carcinoma (RCC), or primary HCC (group B); measurable liver tumors suitable for injection; Eastern Cooperative Oncology Group performance status of 0 or 1; life expectancy of ≥5 months; Child-Pugh score of A to B7; adequate organ function; and receipt of ≥1 prior standard of care systemic therapy for their locally advanced or metastatic disease in patients with non-HCC liver metastases. Patients with melanoma, or NSCLC in combination cohorts were not required to have received prior therapy. Key eligibility criteria in part 2 included having HRBC, TNBC, CSCC, CRC, and BCC with or without liver metastases. With the exception of the CSCC cohort, all patients were required to have received ≥1 prior standard of care therapy for advanced disease.

Primary endpoints were patient incidence of dose-limiting toxicities (DLTs) with T-VEC as monotherapy and in combination with pembrolizumab in part 1; and ORR per irRC-RECIST and patient incidence of treatment-emergent and treatment-related adverse events, including DLTs, with T-VEC+pembrolizumab in part 2. Key secondary endpoints included efficacy, including ORR, best overall response, durable response rate, duration of response, disease control rate (DCR), progression-free survival (PFS), and overall survival (OS), as well as safety.

Safety was assessed for patients who received ≥1 dose of T-VEC monotherapy, or ≥1 dose of T-VEC plus ≥1 dose of pembrolizumab combination therapy. In part 1, safety of T-VEC monotherapy and T-VEC+pembrolizumab combination therapy was assessed based on the 3+3 design and the modified toxicity probability interval (mTPI) up-and-down design, respectively. Interim safety analyses were performed for evaluation of DLTs by a dose level review team. Efficacy was assessed for patients who received ≥1 dose of T-VEC monotherapy, or ≥1 dose of T-VEC plus ≥1 dose of pembrolizumab combination therapy. Patients that initiated treatment at the corresponding RP2D in part 1 were not included in the part 2 efficacy analysis according to their respective tumor type arm. The 2-stage design was used in part 2 to evaluate the ORR per modified irRC-RECIST; a cumulative total of 10 patients will be treated in stage 1 and enrollment is planned to stop. If there are 2 or more responders (PR or CR no confirmation is needed), then a total of 11 additional patients will be treated in stage 2; otherwise, H0 will be accepted and enrollment will be permanently discontinued. If the required number of responders to continue to stage 2 is observed before the end of stage 1 enrollment, then enrollment will not be suspended. H0 will be rejected after stage 2 if there are ≥6 responders (with confirmation) in 21 treated subjects, that is, the observed ORR is ≥28.6%. In the event precisely 21 patients are not included in the analysis in total, H0 will be rejected if the lower limit of the 95% exact binomial CI is >10%. This analysis was done for the TNBC arm only as TNBC arm was the only tumor type to proceed to the second stage. Given only 18 patients with TNBC were treated at the end of stage 2, the Atkinson and Brown method was applied to calculate 95% CI.

Consecutive confirmation of CR, PR, and progressive disease was required within 28 days of the initial assessment, the only exception being when the investigator reported that an initial progressive disease could not be confirmed due to rapid clinical deterioration. Duration of response, PFS, and OS were estimated using the Kaplan-Meier method.

## Drug Information

**Table:** 

Generic/working name	T-VEC, talimogene laherparepvec
Trade name	Imlygic
Company name	Amgen
Drug type	Oncolytic virus
Drug class	Oncologic
Dose	10^6^-10^8^ plaque-forming units/mL up to 4 or 8 mL
Route	Intrahepatic and intratumoral
Schedule of administration	Every 21 days

## Drug Information: Multi-Arm 1

**Table:** 

Generic/working name	Pembrolizumab
Trade name	Keytruda
Company name	Merck & Co., Inc., Rahway, NJ, USA
Drug class	Immune checkpoint inhibitor
Dose	200 mg
Route	Intravenous
Schedule of administration	Every 21 days

## Patient Characteristics: Part 1

**Table:** 

Number of patients, male	46
Number of patients, female	28
Stage	IV
Age: median (range)	61.5 (30-83) years
Number of prior systemic therapies, median (range), T-VEC monotherapy	Group A (*n* = 23): 4.0 (1-9) Group B (*n* = 5): 3.0 (2-4) Total (*N* = 28): 4.0 (1-9)
Number of prior systemic therapies, median (range), T-VEC+pembrolizumab	Group A (*n* = 24): 3.0 (1-6) Group B (*n* = 22): 3.0 (1-5) Total (*N* = 46): 3.0 (1-6)
Performance status: ECOG	0: *n* = 39 1: *n* = 35
Other	In part 1, 23 patients were enrolled into monotherapy group A (non-HCC) and 5 patients were enrolled in monotherapy group B (HCC) (Figures 1 and 2). All 28 patients in the monotherapy groups received T-VEC. A total of 24 patients were enrolled in combination therapy group A (non-HCC) and 22 patients were enrolled in combination therapy group B (HCC). All 46 patients in the combination therapy groups received T-VEC and pembrolizumab. Patients in the monotherapy groups received T-VEC for a median (range) of 6.1 (0.1, 33.1) weeks. Patients in the part 1 combination therapy groups received T-VEC for a median (range) of 9.1 (0.1, 34.1) weeks and pembrolizumab for a median (range) of 4.0 (1, 33) pembrolizumab infusions over 9.2 (0.1, 98.3) weeks. In the monotherapy and combination therapy groups, respectively: 85.7% and 80.4% were White; 64.3% and 84.8% were enrolled from non-USA regions; and 96.4% and 89.1% had received prior anticancer therapy.
Cancer types or histologic subtypes	Non-HCC liver metastasis, *n* = 23; primary HCC; *n* = 5

**Figure 1.**
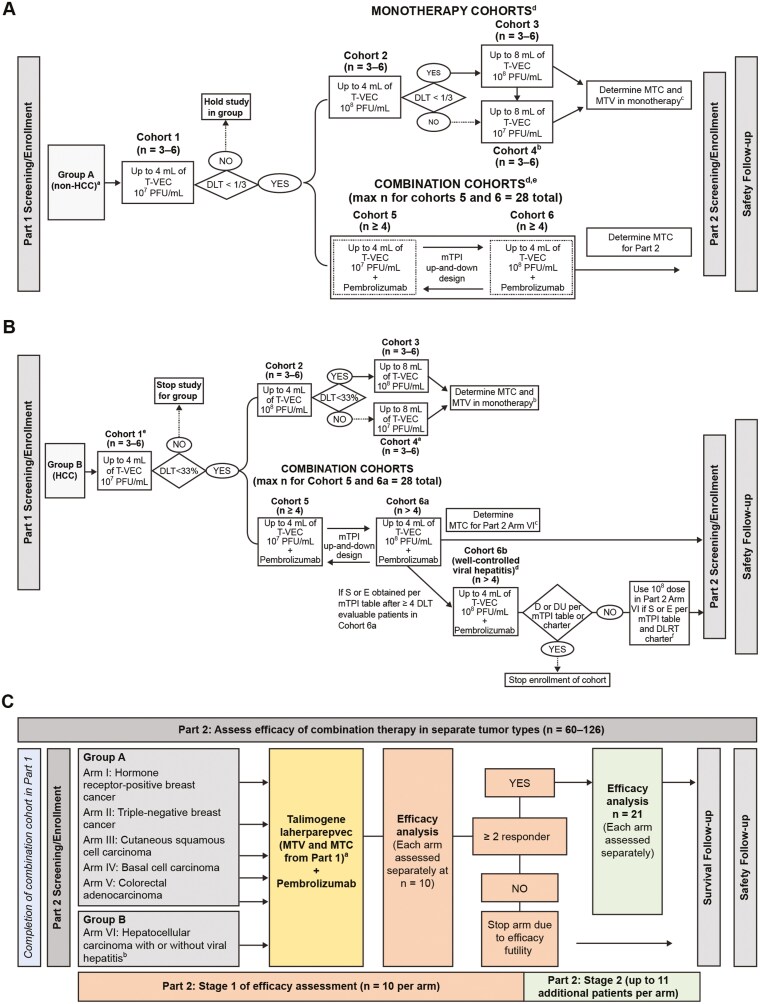
Study design and treatment schema in part 1—group A (A), part 1—group A (B), and part 2 (C). (A) Part 1—group A. ^a^First dose concentration of T-VEC was always 10^6^ PFU/mL. ^b^Cohort 4 was to be opened only if one of these conditions were met: (1) DLT rate ≥1/3 in cohort 2, or (2) DLT rate ≥1/3 in cohort 3 and part 2 dose for T-VEC not determined yet, or (3) DLT rate ≥ 1/3 in cohort 3 and part 2 concentration for T-VEC was determined to be 10^7^ PFU/mL. ^c^MTV determined from monotherapy cohorts, when available, could be used in part 2. ^d^If both cohorts 3 or 4 and the combination cohorts (5 or 6) were open in the same institution, for patients with a tumor burden who could receive 8 mL, enrollment into cohorts 3 or 4 was strongly preferred until the MTV in monotherapy was determined. ^e^Refer to supplemental protocol for mTPI dose decision outcomes. (B) Part 1—group B. ^a^Cohort 4 was to be opened only if cohort 3 T-VEC dose was 10^8^ and (1) DLT > 33% in cohort 3 and part 2 dose for T-VEC not determined yet or (2) DLT > 33% in cohort 3 and part 2 dose for T-VEC is determined to be 10^7^ PFU/mL. ^b^MTV determined from monotherapy cohorts, when available, could be used in part 2 depending on T-VEC combination dose determined for part 2 from cohorts 5 and 6. ^c^If cohort 6b was completed before cohorts 5 and 6a TPI dose finding was completed, and 6b was deemed safe, then cohorts 5 and 6a were to close enrollment and the cohort 6b dose was to be used for Arm VI in part 2. ^d^Cohorts 1-5 and 6a consisted of patients without viral hepatitis, whereas cohort 6b consisted of patients with well-controlled viral hepatitis. ^e^Cohort 1 of group B was to be initiated only after safety had been established in cohort 1 of group A. ^f^If either cohort 6a or 6b in group B of part 1 was deemed unsafe to move to part 2, then only the cohort that was deemed safe was to be enrolled in arm VI in part 2. (C) Part 2 ^a^Could increase up to 8 mL after safety shown in cohort 3 or 4 in part 1. ^b^If either cohort 6a or 6b in group B of part 1 was deemed unsafe to move to part 2, then only the cohort that was deemed safe was to be enrolled in arm VI in part 2. Abbreviations: D, deescalate to the next lower dose; DU, deescalate to the next lower dose/the current dose is unacceptably toxic; DLT, dose-limiting toxicity; DLRT, dose-level review team; E, escalate; HCC, hepatocellular carcinoma; MTC, maximum tolerated concentration; mTPI, modified toxicity probability interval; MTV, maximum tolerated volume; PFU, plaque-forming unit; S, stay at current dose; T‑VEC, talimogene laherparepvec.

**Figure 2.**
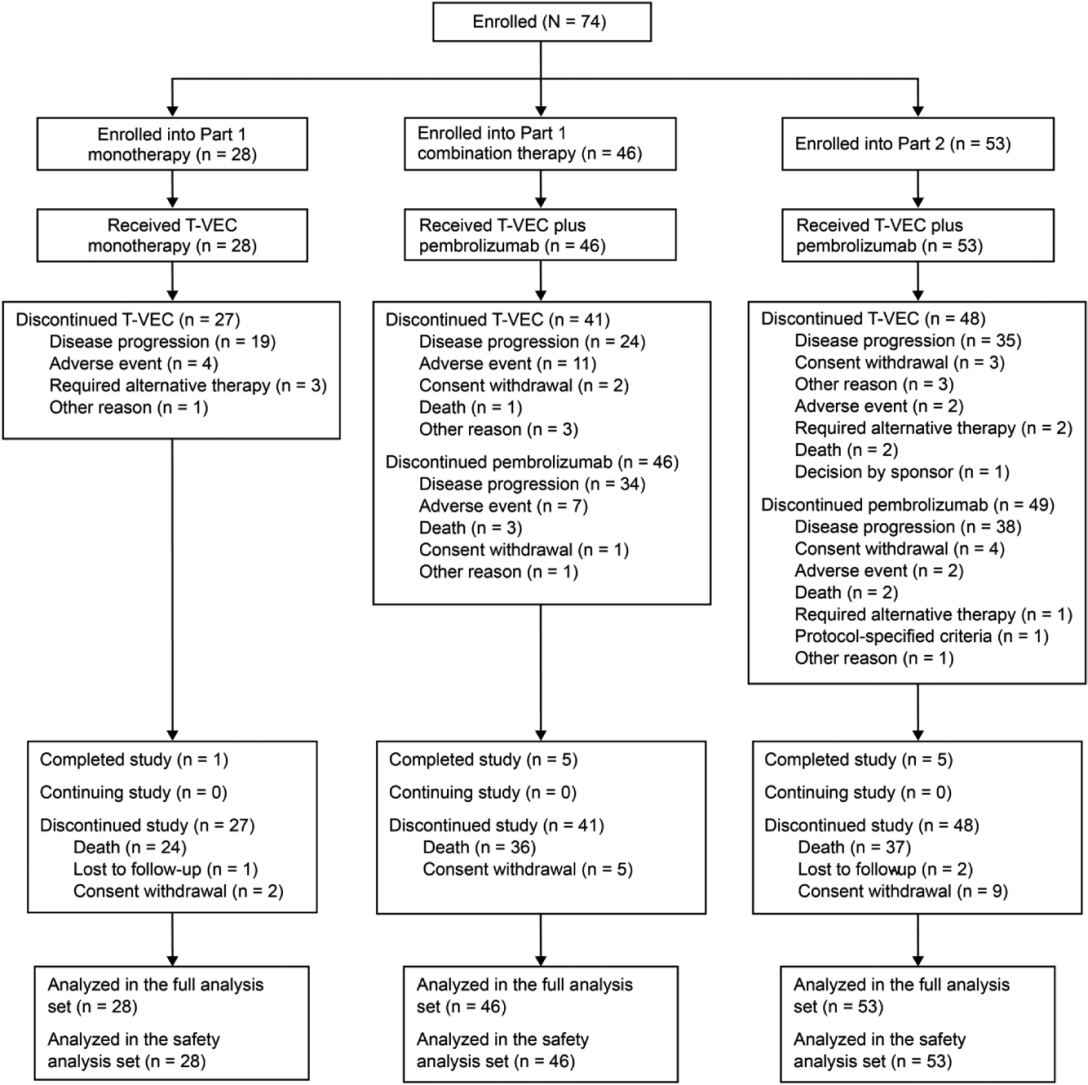
Flow diagram. Abbreviation: T-VEC, talimogene laherparepvec.

## Primary Assessment Method: Trial Profile for Part 1

**Table:** 

Number of patients enrolled	74
Number of patients evaluable for safety	*n* = 28 for T-VEC monotherapy (group A, *n* = 23; group B, *n* = 5) *n* = 46 for T-VEC+pembrolizumab (group A, *n* = 24; group B, *n* = 22)
Number of patients evaluated for efficacy	*n* = 28 for T-VEC monotherapy (group A, *n* = 23; group B, *n* = 5) *n* = 46 for T-VEC+pembrolizumab (group A, *n* = 24; group B, *n* = 22)

Part 1 (*N* = 62) *n*/*N*1 (%). The DLT analysis set included DLT-evaluable patients who have had the opportunity to be on treatment for at least 6 weeks from the initial dosing of study treatment and have received at least 2 doses of T-VEC in monotherapy or at least 2 doses of T-VEC and pembrolizumab in combination, or patients having a DLT during the DLT evaluation period after at least 1 dose of T-VEC in monotherapy or T-VEC and pembrolizumab in combination.

Abbreviations: DLT, dose-limiting toxicity; HCC, hepatocellular carcinoma; *n*, number of patients with DLT; *N*1, number of DLT evaluable patients; T-VEC, talimogene laherparepvec.

## Dose-Limiting Toxicities: Part 1 (DLT Analysis Set)

**Table:** 

T-VEC monotherapy	
Group A (non-HCC)	
Cohort 1	0/6 (0.0)
Cohort 2	1/6 (16.7)
Cohort 3	1/6 (16.7)
Group B (HCC)	
Cohort 1	0/3 (0.0)
Cohort 2	0/2 (0.0)
T-VEC+pembrolizumab	
Group A (non-HCC)	
Cohort 5	1/6 (16.7)
Cohort 6	0/14 (0.0)
Group B (HCC)	
Cohort 5	0/5 (0.0)
Cohort 6a	0/7 (0.0)
Cohort 6b	0/7 (0.0)
Number of patients with T-VEC injection >4 mL	
Group A Cohort 3	1/6 (16.7)

^a^Binomial proportion with exact 95% CI.

The full analysis set included patients who received at least 1 dose of T-VEC in monotherapy cohorts and at least 1 dose of T-VEC and at least 1 dose of pembrolizumab in combination cohorts. ORR is the incidence rate of either a CR or PR based on modified irRC-RECIST criteria.

DCR is the proportion of patients that have a BOR in one of the following: CR/PR/SD. DRR is the rate of patients with an objective response with a DOR of at least 6 months.

Abbreviations: BOR, best overall response; CR, complete response; DCR, disease control rate; DRR, disease response rate; irRC-RECIST, Immune-related Response Criteria Simulating Response Evaluation Criteria in Solid Tumors; ORR, objective response rate; PD, progressive disease; PR, partial response; SD, stable disease; T-VEC, talimogene laherparepvec; UE, unevaluable.

## Response Assessment

**Table:** 

Evaluation method	irRC-RECIST	
Part 1: T-VEC monotherapy	Group A (*N* = 23)	Group B (*N* = 5)
ORR, *n* (% [95% CI^a^])	*n* = 0 (0% [0.0, 14.8])	*n* = 0 (0% [0.0, 52.2])
CR	*n* = 0	*n* = 0
PR	*n* = 0	*n* = 0
SD	*n* = 1 (4.3%)	*n* = 1 (20.0%)
PD	*n* = 7 (30.4%)	*n* = 1 (20.0%)
UE	*n* = 13 (56.5%)	*n* = 3 (60.0%)
DCR, *n* (% [95% CI^a^])	*n* = 1 (4.3% [0.1, 21.9])	*n* = 1 (20.0% [0.5, 71.6])
DRR, *n* (% [95% CI^a^])	*n* = 0 (0% [0.0, 14.8])	*n* = 0 (0% [0.0, 52.2])

Part 1: T-VEC+pembrolizumab	Group A (*N* = 24)	Group B (*N* = 22)
ORR, *n* (% [95% CI^a^])	*n* = 2 (8.3% [1.0, 27.0])	*n* = 3 (13.6% [2.9, 34.9])
CR	*n* = 0	*n* = 0
PR	*n* = 2 (8.3%)	*n* = 3 (13.6%)
SD	*n* = 4 (16.7%)	*n* = 6 (27.3%)
PD	*n* = 10 (41.7%)	*n* = 3 (13.6%)
UE	*n* = 7 (29.2%)	*n* = 10 (45.5%)
DCR, *n* (% [95% CI^a^])	6 (25.0% [9.8, 46.7])	9 (40.9% [20.7, 63.6])
DRR, *n* (% [95% CI^a^])	2 (8.3% [1.0, 27.0])	2 (9.1% [1.1, 29.2])

The full analysis set included patients who received at least 1 dose of T-VEC in monotherapy cohorts and at least 1 dose T-VEC and at least 1 dose of pembrolizumab in combination cohorts.

Abbreviations: HCC, hepatocellular carcinoma; OS, overall survival; PFS, progression-free survival; T-VEC, talimogene laherparepvec.

## Duration Assessments

**Table:** 

	Number	Median, months	95% CI
Part 1: T-VEC monotherapy			
PFS			
Group A (non-HCC)	23	2.3	1.9, 9.0
Group B (HCC)	5	3.9	1.9, NE
OS			
Group A (non-HCC)	23	8.7	2.7, 17.1
Group B (HCC)	5	9.1	3.9, NE

Part 1: T-VEC+pembrolizumab			
PFS			
Group A (non-HCC)	24	2.0	1.9, 6.2
Group B (HCC)	22	8.1	3.2, 13.2
OS			
Group A (non-HCC)	24	7.8	4.4, 14.2
Group B (HCC)	22	12.8	6.8, 29.5

Data are presented as *n* (%) of patients.

TEAEs occurring in ≥10% of patients overall are shown here.

The safety analysis set included all patients who had received at least 1 dose of T-VEC in monotherapy cohorts, and at least 1 dose of T-VEC or at least 1 dose of pembrolizumab in combination cohorts. TEAEs were defined as adverse events with an onset from the first dose of any study drug up to 30 days after the last dose of T-VEC or pembrolizumab, whichever was later.

Adverse events were coded using MedDRA version 26.0.

Abbreviations: ALT, alanine aminotransferase; AST, aspartate aminotransferase; MedDRA, Medical Dictionary for Regulatory Activities, TEAE, treatment-emergent adverse event; T-VEC, talimogene laherparepvec.

## Adverse Events: Part 1

**Table:** 

Any-grade TEAEs	T-VEC monotherapy			T-VEC+pembrolizumab		
	Group A	Group B	Total	Group A	Group B	Total
	(*N* = 23)	(*N* = 5)	(*N* = 28)	(*N* = 24)	(*N* = 22)	(*N* = 46)
Number of patients reporting TEAEs	23 (100.0)	5 (100.0)	28 (100.0)	24 (100.0)	21 (95.5)	45 (97.8)
Pyrexia	19 (82.6)	5 (100.0)	24 (85.7)	22 (91.7)	17 (77.3)	39 (84.8)
Chills	4 (17.4)	1 (20.0)	5 (17.9)	13 (54.2)	8 (36.4)	21 (45.7)
Nausea	6 (26.1)	2 (40.0)	8 (28.6)	14 (58.3)	4 (18.2)	18 (39.1)
Abdominal pain	8 (34.8)	2 (40.0)	10 (35.7)	6 (25.0)	8 (36.4)	14 (30.4)
Fatigue	11 (47.8)	1 (20.0)	12 (42.9)	8 (33.3)	4 (18.2)	12 (26.1)
Vomiting	5 (21.7)	2 (40.0)	7 (25.0)	8 (33.3)	5 (22.7)	13 (28.3)
Anemia	7 (30.4)	0 (0.0)	7 (25.0)	6 (25.0)	3 (13.6)	9 (19.6)
Headache	6 (26.1)	2 (40.0)	8 (28.6)	6 (25.0)	2 (9.1)	8 (17.4)
Abdominal pain upper	4 (17.4)	2 (40.0)	6 (21.4)	6 (25.0)	1 (4.5)	7 (15.2)
Decreased appetite	6 (26.1)	0 (0.0)	6 (21.4)	5 (20.8)	2 (9.1)	7 (15.2)
Pruritus	0 (0.0)	1 (20.0)	1 (3.6)	6 (25.0)	6 (27.3)	12 (26.1)
AST increased	2 (8.7)	0 (0.0)	2 (7.1)	6 (25.0)	3 (13.6)	9 (19.6)
Back pain	4 (17.4)	1 (20.0)	5 (17.9)	5 (20.8)	1 (4.5)	6 (13.0)
Diarrhea	4 (17.4)	0 (0.0)	4 (14.3)	5 (20.8)	2 (9.1)	7 (15.2)
Asthenia	1 (4.3)	2 (40.0)	3 (10.7)	5 (20.8)	2 (9.1)	7 (15.2)
Constipation	5 (21.7)	0 (0.0)	5 (17.9)	4 (16.7)	1 (4.5)	5 (10.9)
Dyspnea	3 (13.0)	3 (60.0)	6 (21.4)	3 (12.5)	1 (4.5)	4 (8.7)
Hypotension	1 (4.3)	1 (20.0)	2 (7.1)	6 (25.0)	2 (9.1)	8 (17.4)
Blood alkaline phosphatase increased	4 (17.4)	2 (40.0)	6 (21.4)	3 (12.5)	0 (0.0)	3 (6.5)
Injection site pain	2 (8.7)	0 (0.0)	2 (7.1)	3 (12.5)	4 (18.2)	7 (15.2)
ALT increased	4 (17.4)	1 (20.0)	5 (17.9)	3 (12.5)	0 (0.0)	3 (6.5)
Platelet count decreased	3 (13.0)	1 (20.0)	4 (14.3)	1 (4.2)	3 (13.6)	4 (8.7)
Rash	2 (8.7)	0 (0.0)	2 (7.1)	4 (16.7)	2 (9.1)	6 (13.0)

Data are presented as *n* (%) of patients.

The safety analysis set included all patients who had received at least 1 dose of T-VEC in monotherapy cohorts, and at least 1 dose of T-VEC or at least 1 dose of pembrolizumab in combination cohorts.

Serious TEAEs were defined as any serious adverse event occurring after initiation of the first dose of study therapy through 90 days after the last administration of study therapy or 30 days after the last administration of study therapy if the patient initiated new anticancer therapy, whichever was earlier.

Adverse events were coded using MedDRA version 26.0.

Abbreviations: ALP, alkaline phosphatase; ALT, alanine aminotransferase; AST, aspartate aminotransferase; MedDRA, Medical Dictionary for Regulatory Activities, TEAE, treatment-emergent adverse event; T-VEC, talimogene laherparepvec.

## Serious Adverse Events: Part 1

**Table:** 

Any grade serious TEAEs	T-VEC monotherapy			T-VEC-pembrolizumab		
	Group A	Group B	Total	Group A	Group B	Total
	(*N* = 23)	(*N* = 5)	(*N* = 28)	(*N* = 24)	(*N* = 22)	(*N* = 46)
Number of patients reporting serious TEAEs	10 (43.5)	2 (40.0)	12 (42.9)	10 (41.7)	11 (50.0)	21 (45.7)
Pyrexia	3 (13.0)	1 (20.0)	4 (14.3)	4 (16.7)	1 (4.5)	5 (10.9)
AST increased	1 (4.3)	0 (0.0)	1 (3.6)	0 (0.0)	1 (4.5)	1 (2.2)
Hepatic hemorrhage	1 (4.3)	0 (0.0)	1 (3.6)	0 (0.0)	1 (4.5)	1 (2.2)
Nausea	1 (4.3)	0 (0.0)	1 (3.6)	0 (0.0)	1 (4.5)	1 (2.2)
Abdominal pain	1 (4.3)	0 (0.0)	1 (3.6)	0 (0.0)	0 (0.0)	0 (0.0)
Acute coronary syndrome	0 (0.0)	0 (0.0)	0 (0.0)	0 (0.0)	1 (4.5)	1 (2.2)
Acute kidney injury	0 (0.0)	0 (0.0)	0 (0.0)	1 (4.2)	0 (0.0)	1 (2.2)
ALT increased	1 (4.3)	0 (0.0)	1 (3.6)	0 (0.0)	0 (0.0)	0 (0.0)
Aspiration	0 (0.0)	0 (0.0)	0 (0.0)	0 (0.0)	1 (4.5)	1 (2.2)
Bacterial infection	1 (4.3)	0 (0.0)	1 (3.6)	0 (0.0)	0 (0.0)	0 (0.0)
Blood ALP increased	0 (0.0)	1 (20.0)	1 (3.6)	0 (0.0)	0 (0.0)	0 (0.0)
Chest pain	0 (0.0)	0 (0.0)	0 (0.0)	0 (0.0)	1 (4.5)	1 (2.2)
Cholestasis	1 (4.3)	0 (0.0)	1 (3.6)	0 (0.0)	0 (0.0)	0 (0.0)
Confusional state	0 (0.0)	0 (0.0)	0 (0.0)	1 (4.2)	0 (0.0)	1 (2.2)
Decreased appetite	0 (0.0)	0 (0.0)	0 (0.0)	0 (0.0)	1 (4.5)	1 (2.2)
Dehydration	1 (4.3)	0 (0.0)	1 (3.6)	0 (0.0)	0 (0.0)	0 (0.0)
Dyspnea	0 (0.0)	0 (0.0)	0 (0.0)	1 (4.2)	0 (0.0)	1 (2.2)
Fatigue	0 (0.0)	0 (0.0)	0 (0.0)	1 (4.2)	0 (0.0)	1 (2.2)
Glomerulonephritis proliferative	0 (0.0)	0 (0.0)	0 (0.0)	1 (4.2)	0 (0.0)	1 (2.2)
Hematoma	0 (0.0)	0 (0.0)	0 (0.0)	1 (4.2)	0 (0.0)	1 (2.2)
Hematuria	0 (0.0)	0 (0.0)	0 (0.0)	0 (0.0)	1 (4.5)	1 (2.2)
Hemorrhage	0 (0.0)	0 (0.0)	0 (0.0)	1 (4.2)	0 (0.0)	1 (2.2)
Hepatic cirrhosis	0 (0.0)	0 (0.0)	0 (0.0)	0 (0.0)	1 (4.5)	1 (2.2)
Hepatitis cholestatic	0 (0.0)	0 (0.0)	0 (0.0)	1 (4.2)	0 (0.0)	1 (2.2)
Hernial eventration	1 (4.3)	0 (0.0)	1 (3.6)	0 (0.0)	0 (0.0)	0 (0.0)
Liver carcinoma ruptured	0 (0.0)	0 (0.0)	0 (0.0)	0 (0.0)	1 (4.5)	1 (2.2)
Multiple organ dysfunction syndrome	0 (0.0)	0 (0.0)	0 (0.0)	0 (0.0)	1 (4.5)	1 (2.2)
Pericarditis	0 (0.0)	0 (0.0)	0 (0.0)	0 (0.0)	1 (4.5)	1 (2.2)
Pneumothorax	1 (4.3)	0 (0.0)	1 (3.6)	0 (0.0)	0 (0.0)	0 (0.0)
Procalcitonin increased	0 (0.0)	0 (0.0)	0 (0.0)	0 (0.0)	1 (4.5)	1 (2.2)
Soft tissue hemorrhage	0 (0.0)	0 (0.0)	0 (0.0)	0 (0.0)	1 (4.5)	1 (2.2)
Spinal cord compression	0 (0.0)	0 (0.0)	0 (0.0)	1 (4.2)	0 (0.0)	1 (2.2)
Syncope	1 (4.3)	0 (0.0)	1 (3.6)	0 (0.0)	0 (0.0)	0 (0.0)
Transaminases increased	0 (0.0)	1 (20.0)	1 (3.6)	0 (0.0)	0 (0.0)	0 (0.0)
Weight decreased	0 (0.0)	0 (0.0)	0 (0.0)	0 (0.0)	1 (4.5)	1 (2.2)

## Patient Characteristics: Part 2

**Table:** 

Patient characteristics	Cohort name: part 2 group A				
Number of patients, male	16				
Number of patients, female	37				
Stage	IV				
Age: median (range)	53.0 (31, 79) years				
Number of prior systemic therapies, median (range)	Arm 1: HRBC (*N* = 10)	Arm 2: TNBC (*N* = 18)	Arm 3: CSCC (*N* = 10)	Arm 4: BCC (*N* = 5)	Arm 5: CRC (*N* = 10)
	4.0 (2-8)	4.0 (2-11)	2.0 (1-4)	2.0 (1-3)	3.0 (2-5)
Performance status: ECOG	0: *n* = 22				
	1: *n* = 31				
Other	In part 2, 53 patients were enrolled across arms 1 through 5. Overall, 84.9% of patients were White, 92.5% were enrolled from non-US regions, and 92.5% had received prior anticancer therapy. All 53 patients received T-VEC and pembrolizumab. Patients received T-VEC for a median (range) of 6.1 (0.1, 102.4) weeks and pembrolizumab for a median (range) of 6.1 (0.1, 109.3) weeks. Group A included patients with advanced triple-negative breast cancer (TNBC), hormone receptor-positive breast cancer (HRBC), colorectal adenocarcinoma (CRC), cutaneous squamous cell carcinoma (CSCC), and basal cell carcinoma (BCC); further details are provided below. Group B was planned to include patients with hepatocellular carcinoma with and without viral hepatitis but did not open for enrolment				
Cancer types or histologic subtypes	HRBC: arm 1, *n* = 10; TNBC: arm 2, *n* = 18; CSCC: arm 3, *n* = 10; BCC: arm 4, *n* = 5; CRC: arm 5, *n* = 10				

## Primary Assessment Method: Trial Profile for Part 2

**Table:** 

Number of patients enrolled	53
Number of patients evaluable for safety	53
Number of patients evaluated for efficacy	53

## Dose Escalation: Parts 1 and 2

**Table:** 

Dose level	Cohort	Dose of drug: T-VEC	Dose of drug: pembrolizumab	Number enrolled	Number evaluable for toxicity	Number of patients with DLT
1	Part 1 group A cohort 1	4 mL × 10^7^ PFU/mL	Not applicable	7	6	0
2	Part 1 group A cohort 2	4 mL × 10^8^ PFU/mL	Not applicable	7	6	1
3	Part 1 group A cohort 3	8 mL × 10^8^ PFU/mL	Not applicable	9	6	1
4	Part 1 group A cohort 5	4 mL × 10^7^ PFU/mL	200 mg	7	6	1
5	Part 1 group A cohort 6	4 mL × 10^8^ PFU/mL	200 mg	17	14	0
6	Part 1 group B cohort 1	4 mL × 10^7^ PFU/mL	Not applicable	3	3	0
7	Part 1 group B cohort 2	4 mL × 10^8^ PFU/mL	Not applicable	2	2	0
8	Part 1 group B cohort 5	4 mL × 10^7^ PFU/mL	200 mg	5	5	0
9	Part 1 group B cohorts 6a+6b	4 mL × 10^8^ PFU/mL	200 mg	17	14	0
10	Part 2 group A arm 1	8 mL × 10^8^ PFU/mL	200 mg	10	8	1
11	Part 2 group A arm 2	8 mL × 10^8^ PFU/mL	200 mg	18	11	0
12	Part 2 group A arm 3	8 mL × 10^8^ PFU/mL	200 mg	10	5	0
13	Part 2 group A arm 4	8 mL × 10^8^ PFU/mL	200 mg	5	4	0
14	Part 2 group A arm 5	8 mL × 10^8^ PFU/mL	200 mg	10	9	0

## Dose Escalation: Parts 1 and 2 (Combined Cohorts at Each Dose Level)

**Table:** 

Dose level	Dose of drug: T-VEC	Dose of drug: pembrolizumab	Number enrolled	Number evaluable for toxicity	Number of patients with DLT
1	4 mL × 10^7^ PFU/mL	Not applicable	10	9	0
2	4 mL × 10^7^ PFU/mL	200 mg	12	11	1
3	4 mL × 10^8^ PFU/mL	Not applicable	9	8	1
4	4 mL × 10^8^ PFU/mL	200 mg	34	28	0
5	8 mL × 10^8^ PFU/mL	Not applicable	9	6	1
6	8 mL × 10^8^ PFU/mL	200 mg	53	37	1

The DLT analysis set included DLT-evaluable patients who have had the opportunity to be on treatment for at least 6 weeks from the initial dosing of study treatment and had received at least 2 doses of T-VEC and 2 doses of pembrolizumab in combination, or had a DLT during the DLT evaluation period after at least 1 dose of T-VEC and pembrolizumab in combination. Both weeks 1 and 4 injections are considered for hepatic and non-hepatic lesion injection sites.

Abbreviations: BCC, basal cell carcinoma; CRC, colorectal adenocarcinoma; CSCC, cutaneous squamous cell carcinoma; DLT, dose-limiting toxicity; HCC, hepatocellular carcinoma; HRBC, hormone receptor positive breast adenocarcinoma. *n* = number of patients with DLT; *m* = number of DLT evaluable patients; TNBC, triple-negative breast cancer; T-VEC, talimogene laherparepvec.

## Dose-Limiting Toxicities: Part 2

**Table:** 

Part 2 group A (non-HCC)	*N* = 37 n/m (%)
Arm 1: HRBC	1/8 (12.5)
Injection site	
Both hepatic and non-hepatic	0/0 (—)
Hepatic only	1/4 (25.0)
Non-hepatic only	0/4 (0.0)
Arm 2: TNBC	0/11 (0.0)
Injection site	
Both hepatic and non-hepatic	0/1 (0.0)
Hepatic only	0/2 (0.0)
Non-hepatic only	0/8 (0.0)
Arm 3: CSCC	0/5 (0.0)
Injection site	
Both hepatic and non-hepatic	0/0 (—)
Hepatic only	0/0 (—)
Non-hepatic only	0/5 (0.0)
Arm 4: BCC	0/4 (0.0)
Injection site	
Both hepatic and non-hepatic	0/0 (—)
Hepatic only	0/0 (—)
Non-hepatic only	0/4 (0.0)
Arm 5: CRC	0/9 (0.0)
Injection site	
Both hepatic and non-hepatic	0/0 (—)
Hepatic only	0/8 (0.0)
Non-hepatic only	0/1 (0.0)
Number of patients with T-VEC injection >4 mL	0/11 (0.0)
Arm 1: HRBC	0/2 (0.0)
Arm 2: TNBC	0/5 (0.0)
Arm 3: CSCC	0/4 (0.0)
Arm 4: BCC	0/0 (—)
Arm 5: CRC	0/0 (—)

Abbreviations: BCC, basal cell carcinoma; CR, complete response; CRC, colorectal adenocarcinoma; CSCC, cutaneous squamous cell carcinoma; DCR, disease control rate; DRR, disease response rate; HCC, hepatocellular carcinoma; HRBC, hormone receptor positive breast adenocarcinoma; irRC-RECIST, Immune-related Response Criteria simulating Response Evaluation Criteria in Solid Tumors; ORR, objective response rate; PD, progressive disease; PR, partial response; SD, stable disease; TNBC, triple-negative breast cancer; UE, unevaluable.

## Response Assessment: Part 2

**Table:** 

	irRC-RECIST				
	Arm 1: HRBC (*N* = 10)	Arm 2: TNBC (*N* = 18)	Arm 3: CSCC (*N* = 10)	Arm 4: BCC (*N* = 5)	Arm 5: CRC (*N* = 10)
ORR, *n* (% [95% CI^a^]	*n* = 1 (10.0% [0.3, 44.5])	*n* = 3 (16.7% [3.6, 41.4])	*n* = 1 (10.0% [0.3, 44.5])	*n* = 1 (20.0% [0.5, 71.6])	*n* = 0 (0% [0.0, 30.8])
CR	*n* = 0	*n* = 2 (11.1%)	*n* = 0	*n* = 0	*n* = 0
PR	*n* = 1 (10.0%)	*n* = 1 (5.6%)	*n* = 1 (10.0%)	*n* = 1 (20.0%)	*n* = 0
SD	*n* = 1 (10.0%)	*n* = 1 (5.6%)	*n* = 1 (10.0%)	*n* = 2 (40.0%)	*n* = 3 (30.0%)
PD	*n* = 3 (30.0%)	*n* = 5 (27.8%)	*n* = 2 (20.0%)	*n* = 0	*n* = 1 (10.0%)
UE	*n* = 5 (50.0%)	*n* = 6 (33.3%)	*n* = 4 (40.0%)	*n* = 2 (40.0%)	*n* = 5 (50.0%)
DCR, *n* (%[95% CI^a^])	*n* = 2 (20.0% [2.5, 55.6])	*n* = 4 (22.2% [6.4, 47.6])	*n* = 2 (20.0% [2.5, 55.6])	*n* = 3 (60.0% [14.7, 94.7])	*n* = 3 (30.0% [6.7, 65.2])
DRR, *n* (%[95% CI^a^])	*n* = 1 (10.0% [0.3, 44.5])	*n* = 2 (11.1% [1.4, 34.7])	*n* = 1 (10.0% [0.3, 44.5])	*n* = 1 (20.0% [0.5, 71.6])	*n* = 0 (0% [0.0, 30.8])

^a^Binomial proportion with exact 95% CI.

The full analysis set included patients who received at least 1 dose T-VEC and at least 1 dose of pembrolizumab. OS is defined as the time from the date of first dose date to the date of death from any cause.

Only one solid tumor cohort (TNBC) enrolled more than 10 patients due to lack of efficacy and fulfillment of futility analysis in other cohorts. ORR, duration of response, PFS, and OS in TNBC was limited, with no apparent benefit to the use of T-VEC and pembrolizumab combination.

Group B (HCC) did not open for enrolment.

Abbreviations: BCC, basal cell carcinoma; CRC, colorectal adenocarcinoma; CSCC, cutaneous squamous cell carcinoma; HCC, hepatocellular carcinoma; HRBC, hormone receptor positive breast adenocarcinoma; irRC-RECIST, Immune-related Response Criteria simulating Response Evaluation Criteria in Solid Tumors; NE, not estimable; OS, overall survival; PFS, progression-free survival; TNBC, triple-negative breast cancer.

## Duration Assessments: Part 2

**Table:** 

	Number	Median, months	95% CI
PFS			
Arm 1: HRBC	10	6.1	1.6, 20.5
Arm 2: TNBC	18	2.9	1.2, 10.2
Arm 3: CSCC	10	5.4	2.2, NE
Arm 4: BCC	5	16.4	5.4, NE
Arm 5: CRC	10	8.8	2.4, 12.5
OS			
Arm 1: HRBC	10	9.1	2.4, 20.5
Arm 2: TNBC	18	10.2	2.7, 27.2
Arm 3: CSCC	10	9.6	2.3, NE
Arm 4: BCC	5	16.4	5.4, NE
Arm 5: CRC	10	11.2	2.4, 18.9

Data are presented as *n* (%) of patients.

TEAEs occurring in ≥10% of patients overall are shown here.

The safety analysis set included all patients who had received at least 1 dose of T-VEC or at least 1 dose of pembrolizumab.

TEAEs were defined as adverse events with an onset from the first dose of any study drug up to 30 days after the last dose of T-VEC or pembrolizumab, whichever was later.

Adverse events were coded using MedDRA version 26.0.

Abbreviations: BCC, basal cell carcinoma; CRC, colorectal adenocarcinoma; CSCC, cutaneous squamous cell carcinoma; HRBC, hormone receptor positive breast adenocarcinoma; MedDRA, Medical Dictionary for Regulatory Activities; TEAE, treatment-emergent adverse event; TNBC, triple-negative breast cancer.

## Adverse Events: Part 2

**Table:** 

Any-grade TEAEs	Arm 1: HRBC (*N* = 10)	Arm 2: TNBC (*N* = 18)	Arm 3: CSCC (*N* = 10)	Arm 4: BCC (*N* = 5)	Arm 5: CRC (*N* = 10)
Number of patients reporting TEAEs	10 (100.0)	17 (94.4)	9 (90.0)	5 (100.0)	10 (100.0)
Pyrexia	10 (100.0)	10 (55.6)	3 (30.0)	1 (20.0)	10 (100.0)
Nausea	7 (70.0)	3 (16.7)	2 (20.0)	1 (20.0)	4 (40.0)
Chills	8 (80.0)	0 (0.0)	2 (20.0)	0 (0.0)	3 (30.0)
Fatigue	3 (30.0)	1 (5.6)	1 (10.0)	2 (40.0)	5 (50.0)
Vomiting	5 (50.0)	3 (16.7)	1 (10.0)	0 (0.0)	2 (20.0)
Anemia	2 (20.0)	3 (16.7)	1 (10.0)	2 (40.0)	2 (20.0)
Asthenia	3 (30.0)	5 (27.8)	0 (0.0)	0 (0.0)	2 (20.0)
Injection site pain	2 (20.0)	1 (5.6)	2 (20.0)	0 (0.0)	4 (40.0)
Arthralgia	3 (30.0)	2 (11.1)	1 (10.0)	1 (20.0)	1 (10.0)
Decreased appetite	3 (30.0)	1 (5.6)	0 (0.0)	1 (20.0)	3 (30.0)
Dyspnea	3 (30.0)	2 (11.1)	1 (10.0)	1 (20.0)	1 (10.0)
Rash	1 (10.0)	1 (5.6)	3 (30.0)	2 (40.0)	1 (10.0)
Hypotension	3 (30.0)	3 (16.7)	1 (10.0)	0 (0.0)	0 (0.0)
Blood alkaline phosphatase increased	3 (30.0)	2 (11.1)	0 (0.0)	0 (0.0)	1 (10.0)
Constipation	3 (30.0)	0 (0.0)	0 (0.0)	2 (40.0)	1 (10.0)
Diarrhea	4 (40.0)	0 (0.0)	1 (10.0)	1 (20.0)	0 (0.0)

Data are presented as *n* (%) of patients.

The safety analysis set included all patients who had received at least 1 dose of T-VEC or at least 1 dose of pembrolizumab.

Serious TEAEs were defined as any serious adverse event occurring after initiation of the first dose of study therapy through 90 days after the last administration of study therapy or 30 days after the last administration of study therapy if the patient initiated new anticancer therapy, whichever was earlier.

Adverse events were coded using MedDRA version 26.0.

Abbreviations: BCC, basal cell carcinoma; CRC, colorectal adenocarcinoma; CSCC, cutaneous squamous cell carcinoma; HRBC, hormone receptor-positive breast adenocarcinoma; MedDRA, Medical Dictionary for Regulatory Activities; TEAE, treatment-emergent adverse event; TNBC, triple-negative breast cancer.

## Serious Adverse Events: Part 2

**Table:** 

Any-grade serious TEAEs	Arm 1: HRBC (*N* = 10)	Arm 2: TNBC (*N* = 18)	Arm 3: CSCC (*N* = 10)	Arm 4: BCC (*N* = 5)	Arm 5: CRC (*N* = 10)
Number of patients reporting serious TEAEs	4 (40.0)	7 (38.9)	5 (50.0)	3 (60.0)	3 (30.0)
Pyrexia	2 (20.0)	2 (11.1)	0 (0.0)	0 (0.0)	1 (10.0)
Dyspnea	0 (0.0)	2 (11.1)	1 (10.0)	0 (0.0)	0 (0.0)
Hypotension	2 (20.0)	1 (5.6)	0 (0.0)	0 (0.0)	0 (0.0)
Acute kidney injury	0 (0.0)	1 (5.6)	0 (0.0)	0 (0.0)	1 (10.0)
Tumor pain	0 (0.0)	0 (0.0)	1 (10.0)	1 (20.0)	0 (0.0)
Abdominal pain	0 (0.0)	0 (0.0)	0 (0.0)	0 (0.0)	1 (10.0)
Aphasia	0 (0.0)	1 (5.6)	0 (0.0)	0 (0.0)	0 (0.0)
Breast cancer metastatic	0 (0.0)	1 (5.6)	0 (0.0)	0 (0.0)	0 (0.0)
Confusional state	0 (0.0)	1 (5.6)	0 (0.0)	0 (0.0)	0 (0.0)
Cytokine release syndrome	0 (0.0)	1 (5.6)	0 (0.0)	0 (0.0)	0 (0.0)
Decubitus ulcer	0 (0.0)	0 (0.0)	1 (10.0)	0 (0.0)	0 (0.0)
Diabetic ketoacidosis	1 (10.0)	0 (0.0)	0 (0.0)	0 (0.0)	0 (0.0)
Hemophilus infection	0 (0.0)	0 (0.0)	1 (10.0)	0 (0.0)	0 (0.0)
Hepatic hemorrhage	0 (0.0)	1 (5.6)	0 (0.0)	0 (0.0)	0 (0.0)
Hypercalcemia	0 (0.0)	1 (5.6)	0 (0.0)	0 (0.0)	0 (0.0)
Hypovolemic shock	1 (10.0)	0 (0.0)	0 (0.0)	0 (0.0)	0 (0.0)
Ileus	0 (0.0)	0 (0.0)	1 (10.0)	0 (0.0)	0 (0.0)
Intestinal perforation	0 (0.0)	1 (5.6)	0 (0.0)	0 (0.0)	0 (0.0)
Iron deficiency anemia	0 (0.0)	1 (5.6)	0 (0.0)	0 (0.0)	0 (0.0)
Metastases to lung	0 (0.0)	0 (0.0)	0 (0.0)	1 (20.0)	0 (0.0)
Muscular weakness	1 (10.0)	0 (0.0)	0 (0.0)	0 (0.0)	0 (0.0)
Pain in extremity	0 (0.0)	0 (0.0)	0 (0.0)	0 (0.0)	1 (10.0)
Peritonitis	0 (0.0)	0 (0.0)	0 (0.0)	0 (0.0)	1 (10.0)
Pleural effusion	0 (0.0)	0 (0.0)	0 (0.0)	1 (20.0)	0 (0.0)
Pseudomonal skin infection	0 (0.0)	0 (0.0)	0 (0.0)	1 (20.0)	0 (0.0)
Respiratory tract infection	0 (0.0)	0 (0.0)	1 (10.0)	0 (0.0)	0 (0.0)
Soft tissue infection	0 (0.0)	0 (0.0)	1 (10.0)	0 (0.0)	0 (0.0)
Urinary retention	0 (0.0)	0 (0.0)	1 (10.0)	0 (0.0)	0 (0.0)
Urinary tract infection bacterial	0 (0.0)	1 (5.6)	0 (0.0)	0 (0.0)	0 (0.0)

## Assessment, Analysis, and Discussion

**Table:** 

Completion	Study completed
Investigator’s assessment	Poorly tolerated/not feasible; level of activity did not meet planned endpoint

The liver is a frequent site of cancer metastases, in addition to being the organ of origin for hepatocellular carcinoma (HCC). Localized HCC is primarily treated by surgical resection, liver transplantation, or locally ablative therapies.^1^ Liver resection is considered for patients with limited disease in HCC and other solid tumors.^1^ For solid tumors with metastasis to the liver, systemic therapy with chemotherapy, often combined with targeted agents, is the primary treatment modality. Despite available treatment options, unresectable HCC with liver metastases generally portend a poor prognosis. Furthermore, the presence of liver metastases has been shown to diminish immunotherapy efficacy systemically.^2^ In murine models, liver metastases siphon activated CD8+ T cells from the systemic circulation and within the liver, resulting in acquired resistance to immunotherapy.^2^ Collectively, a critical unmet need exists for novel treatment strategies to help overcome immunotherapy resistance in the presence of liver metastases.

This study was initiated at a time when there was a significant lack of clinically effective therapies for this difficult-to-treat patient population. The treatment of HCC has evolved markedly in recent years and while this study was ongoing, with the introduction of several FDA-approved systemic therapies.^1^ While sorafenib was approved in 2007 for unresectable or metastatic HCC, additional approvals of small molecule multi-kinase inhibitors, PD-1/PD-L1 inhibitors, anti-CTLA-4 checkpoint inhibitors, and anti-VEGF monoclonal antibodies occurred from 2017 onwards. Pembrolizumab, a PD-1 inhibitor, received accelerated FDA approval in 2018 for the treatment of patients with advanced HCC previously treated with sorafenib on the basis of the global phase II KEYNOTE-224 study.^3^ Pembrolizumab demonstrated antitumor activity and a manageable safety profile and fulfilled an unmet need in the second-line treatment of HCC, for which resistance to targeted agents is common.^3^

T-VEC is an oncolytic viral immunotherapy designed to selectively replicate within tumors and produce granulocyte-macrophage colony-stimulating factor to induce systemic antitumor immunity with changes to the local tumor microenvironment (TME). These TME alterations suggest a potential for further enhancement of efficacy with checkpoint inhibitors, prompting interest in combining T-VEC with pembrolizumab. The combination of an agent that increases tumor-specific immune activation (T-VEC) with one that blocks inhibitory T-cell checkpoints (pembrolizumab) could potentially yield greater antitumor activity than either agent alone. Despite early phase Ib data suggesting potential increased activity, the randomized phase III MASTERKEY-265 trial that assessed T-VEC+pembrolizumab in treatment-naïve patients with advanced melanoma showed that the combination did not significantly improve PFS or OS compared with placebo-pembrolizumab.^4^ The safety profile of T-VEC+pembrolizumab was consistent with that of either agent alone.^4^

In the current study, only TNBC arm in part 2 enrolled more than 10 patients (*n* = 18). This cohort showed an ORR of 16.7%, with 2 CRs and 1 PR. One additional patient had stable disease, resulting in an overall DCR of 22% with the T-VEC-pembrolizumab combination. The median PFS was 2.9 months and median OS was 10.2 months. It cannot be concluded that the combination provided enhanced efficacy over pembrolizumab alone. In accordance with our data, recent studies that have evaluated T-VEC alone or combined with chemotherapy or checkpoint inhibitors in patients with breast cancer have also shown inconclusive or limited antitumor activity. Kai et al evaluated T-VEC (via intratumoral injection) in patients with inoperable locoregional recurrence of breast cancer. While that study showed unfavorable disease control due to an increase in local tumor burden and/or the occurrence of new distant metastases, the authors concluded that future studies should combine intratumoral T-VEC administration with concurrent systemic therapy for optimal outcome.^5^ This approach was taken in another single-center phase II study in TNBC, where intratumoral T-VEC injection was combined with neoadjuvant chemotherapy.^6^ The primary endpoint was met with an estimated residual cancer burden (RBC) zero rate of 45.9% and a 2-year disease-free rate of 89% with no recurrences in patients with RCB 0-1. An additional phase Ib study was conducted concurrent to the present study to evaluate the combination of intrahepatic T-VEC plus atezolizumab in patients with TNBC and CRC with liver metastases and showed results similar to that seen with intrahepatic T-VEC+pembrolizumab in the present study.^7^ The safety profile reflected known adverse events with T-VEC and atezolizumab, with limited evidence of antitumor activity.

In conclusion, limited evidence of antitumor activity suggests that intrahepatic injection of T-VEC with or without pembrolizumab is not recommended for treatment of solid tumors with liver metastases and primary HCC.

**Figure 3.**
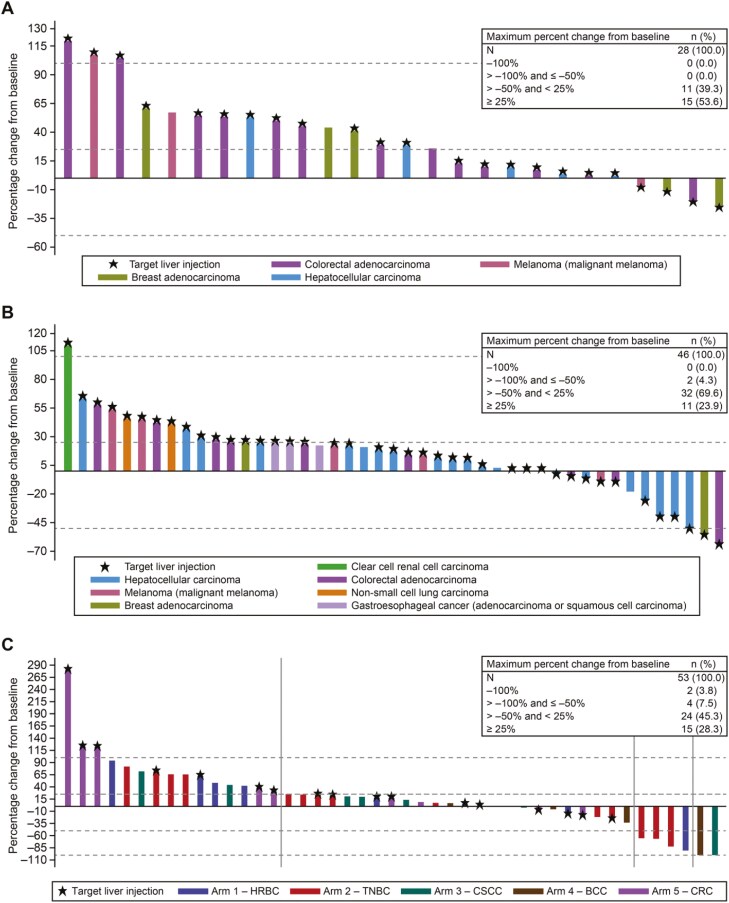
Waterfall plots depicting maximum percent change from baseline in measurable tumor burden by patient for (A) part 1 T-VEC monotherapy group, (B) part 1 T-VEC+pembrolizumab group, and (C) part 2. Maximum decrease in measurable tumor burden by patient. Abbreviations: BCC, basal cell carcinoma; CRC, colorectal adenocarcinoma; CSCC, cutaneous squamous cell carcinoma; HRBC, hormone receptor-positive breast adenocarcinoma; TNBC, triple-negative breast cancer.

## Data Availability

Qualified researchers may request data from Amgen clinical studies. Complete details are available at the following: http://www.amgen.com/datasharing.

## Previous presentation

ASCO 2020—Abstract #3015 and poster; Title: Early Safety From a phase I, Multicenter, Open-label Clinical Trial of Talimogene Laherparepvec (T-VEC) Injected Into Liver Tumors in Combination With Pembrolizumab. ESMO Immuno-Oncology Congress; Geneva, Switzerland, 2021; Title: Biomarker Analysis From a Phase 1b, Multicenter, Open-label Clinical Trial of Talimogene Laherparepvec (T-VEC) Injected Into Metastatic and Primary Liver Tumors.
